# Umbribacter vaginalis gen. nov., sp. nov.: novel bacterium isolated from the human vagina

**DOI:** 10.1099/ijsem.0.006931

**Published:** 2025-10-03

**Authors:** Sujatha Srinivasan, Susan M. Strenk, May A. Beamer, Tina L. Fiedler, Sean Proll, Gabriela R. Acevedo-Oquendo, Gina M. Bonura, G. A. Nagana Gowda, Daniel Raftery, Sharon L. Hillier, David N. Fredricks

**Affiliations:** 1 Vaccine and Infectious Disease Division, Fred Hutchinson Cancer Center, Seattle, WA, USA; 2 Magee-Womens Research Institute, Pittsburgh, PA, USA; 3 Northwest Metabolomics Research Center and Mitochondrial and Metabolism Center, Department of Anesthesiology and Pain Medicine, University of Washington Medical Center, Seattle, WA, USA; 4 Public Health Sciences Division, Fred Hutchinson Cancer Center, Seattle, WA, USA; 5 University of Pittsburgh School of Medicine, Department of Obstetrics, Gynecology and Reproductive Sciences, Pittsburgh, PA, USA; 6 Department of Medicine, University of Washington, Seattle, WA, USA

**Keywords:** bacterial vaginosis, bacterial vaginosis (BV)-associated bacteria, *Eggerthellaceae*, genital tract, human vagina

## Abstract

Gram-variable obligately anaerobic novel bacteria DNF00809 and PR-HUZ-602407-17 were isolated from vaginal fluid samples from women with bacterial vaginosis (BV) in two independent studies conducted in different laboratories. They each displayed ≥99.9% 16S rRNA gene sequence identity to the uncultured bacterial clone sequence AY738656 designated as *Eggerthella*-like vaginal bacterium (ELVB) and shared 100% 16S rRNA gene sequence identity with each other. Studies using molecular bacterial identification have associated ELVB sequences with BV, higher risk for human immunodeficiency virus acquisition and development of pelvic inflammatory disease in women. Given the clinical significance of this bacterium, we characterized the novel bacterium designated DNF00809^T^ using biochemical, genotypic and phylogenetic analyses. DNF00809^T^ was a coccobacillus that was non-motile, non-spore forming, asaccharolytic, proteolytic and indole negative. Fatty acid methyl ester analysis for DNF00809^T^ indicated C_14 : 0_, C_16 : 0_, C_16 : 0_ dimethyl acetal and C_18 : 1_*cis*9 to be the major fatty acids. Whole genomic DNA G+C content was 46.1 mol%. Phylogenetic and phylogenomic analyses indicate that DNF00809^T^ represents a novel genus and novel species within the *Eggerthellaceae* family. We propose the name *Umbribacter vaginalis* gen. nov., sp. nov. with DNF00809^T^ representing the type strain of this species (=DSM 118866^T^=CCUG77988^T^).

## Introduction

Novel anaerobic vaginal bacterial strains DNF00809 and PR-HUZ-602407-17 isolated from human vaginal fluid samples in two separate studies showed ≥99.9% 16S rRNA gene sequence identity to the uncultured bacterial clone sequence AY738656 designated as *Eggerthella*-like vaginal bacterium (ELVB) first identified by cultivation-independent methods to characterize the vaginal microbiota [1]. The 16S rRNA gene sequences of both isolates were 100% identical with each other. These ELVB bacterial clone sequences have been shown to be associated with bacterial vaginosis (BV) in several studies [1,5]. Moreover, ELVB sequences were found to be highly sensitive (89%) and specific (95%) for diagnosing BV using targeted PCR [6]. Interestingly, ELVB was associated with all four individual Amsel clinical criteria used for diagnosing BV (vaginal discharge, an amine odour, presence of >20% clue cells and an elevated pH >4.5) in a Seattle population, and all criteria but discharge among US and Kenyan women participating in the Preventing Vaginal Infections trial [5,7]. In a longitudinal study evaluating the response of individual vaginal bacteria to metronidazole therapy, concentrations of bacteria were measured daily for 15 days. ELVB tended to persist beyond 7 days, suggesting that a 5-to-7-day treatment course of metronidazole may not be sufficient to fully suppress ELVB *in vivo* [8].

In addition to strong associations with BV, ELVB has been associated with a tenfold increased risk for developing subsequent pelvic inflammatory disease (PID) among individuals that tested positive for ELVB in the preceding 3 months of PID diagnosis [9]. Concentrations of ELVB have also been associated with increased risk for acquiring human immunodeficiency virus (HIV) among East and Southern African women [10,11]. Extra-vaginally, ELVB has been detected in the anus and labium and occasionally in the mouth [12]. ELVB sequences have been noted in cervical samples and, in rare instances, fallopian tube and ovary samples, suggesting that ELVB can ascend the genital tract [13].

Although we have limited understanding of the mechanistic role of ELVB in BV and how they mediate PID or HIV risk, their clinical significance warrants systematic characterization and valid naming of this novel vaginal bacterium as it will enable reproducible research using a well-characterized type strain across laboratories. Here, we compare the ELVB isolate DNF00809 with validly named species within the *Eggerthellaceae* family. We propose the name *Umbribacter vaginalis* gen. nov., sp. nov. for DNF00809^T^ which represents the type strain.

## Isolation

DNF00809^T^ was isolated from a vaginal swab sample from a woman attending a Sexual Health Clinic in Seattle who was positive for BV [14]. The original study sought to isolate and characterize novel bacterial isolates from the human vagina and was approved by the Fred Hutchinson Cancer Center Institutional Review Board (IRB approval number: IR7363). The vaginal swab was transported to the clinic within 2 h of collection and processed in the laboratory within 4 h to maximize recovery of bacterial isolates. The swab sample was vortexed in 1.5 ml of reduced Hank’s solution [15], and the resulting vaginal fluid was serially diluted in reduced saline. Aliquots (100 µl) from a range of dilutions were inoculated onto Brucella blood agar with hemin and vitamin K (BRU) (Hardy Diagnostics, Santa Maria, CA) and incubated under anaerobic conditions at 37 °C for 5 days. A colony that was tiny, transparent (almost like a water droplet) and entire with a raised centre was picked from the 10^−7^ dilution plate and plated on Brucella agar for further workup. Cells were Gram-variable cocci and coccobacilli, while 16S rRNA sequencing indicated a novel *Eggerthellaceae* bacterium with a 16S rRNA gene sequence identity >99.9% to the uncultured ELVB bacterial clone sequence AY738656. For some experiments, we included a second strain, PR-HUZ-602407-17, isolated on BRU agar at 10^−5^ dilution in the same manner as described earlier from a vaginal fluid sample collected from a pregnant participant who was BV positive in a study protocol approved by the University of Pittsburgh IRB (IRB approval number: PRO15080354). Gram staining of the isolate PR-HUZ-602407-17 indicated small Gram-positive cocci that sometimes occurred in chains, and 16S rRNA gene sequencing indicated ≥99.9% sequence similarity with the ELVB clone sequence AY738656. Study participants from both studies provided written informed consent.

## 16S rRNA gene sequence analysis

**Fig. 1.**
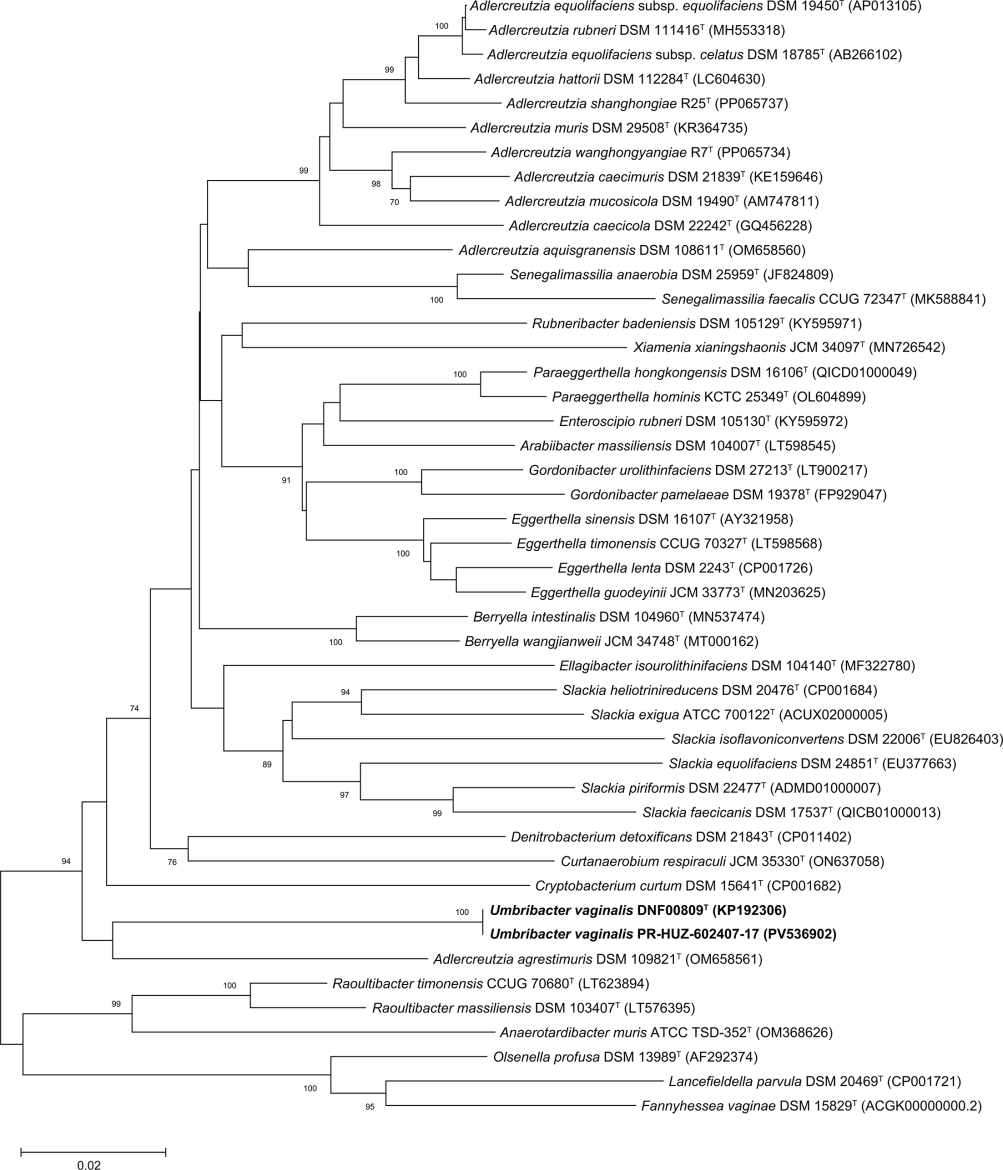
Molecular phylogenetic analysis by maximum composite likelihood method based on 16S rRNA gene sequences showing the phylogenetic positions of *U. vaginalis* DNF00809^T^ and *U. vaginalis* PR-HUZ-602407-17 in comparison with validly named members of the family *Eggerthellaceae*. Bootstrap values (based on 1000 replications) greater than or equal to 70% are shown as percentages at each node. Bar, 0.02 substitutions per nt position. *Olsenella profusa* DSM 13989^T^ (AF292374), *Lancefieldella parvula* DSM 20469^T^ (NR_102936) and *Fannyhessea vaginae* DSM 15829^T^ (ACGK00000000.2) from the family *Atopobiaceae* were added as the outgroup.

The 16S rRNA gene sequences for DNF00809^T^ and PR-HUZ602407-17 were obtained by Sanger sequencing of the 16S rRNA gene. NCBI blast searches (https://blast.ncbi.nlm.nih.gov/Blast.cgi) [16] of the DNF00809^T^ and PR-HUZ602407-17 16S rRNA gene sequences resulted in matches to the uncultivated clone sequence AY738656 designated uncultured *Eggerthella*-like vaginal bacterial clone 123-f2 [1] from the human vagina with a ≥99.9% sequence identity. The 16S rRNA genes of DNF00809^T^ and PR-HUZ602407-17 had a sequence identity of 100% when compared with each other despite being cultivated in two separate studies. These sequences were compared with 16S rRNA gene sequences of other validly named members of the *Eggerthellaceae* (Fig. 1). A multiple sequence alignment of the 16S rRNA genes from the vaginal isolates and other validly named type strains from the *Eggerthellaceae* family was created using the clustalw algorithm, and evolutionary relationships were inferred by using the maximum composite likelihood method based on the Tamura–Nei model [17] in megax [18]. The closest neighbour of DNF00809^T^ was PR-HUZ602407-17, and both strains were phylogenetically distinct from the only other type strain in the same clade, *Adlercreutzia agrestimuris* DSM 109821^T^ (Fig. 1). Sequence identities of the 16S rRNA genes from DNF00809^T^ and PR-HUZ602407-17 were 91.6% when compared with the 16S rRNA gene of *A. agrestimuris*, suggesting that these isolates represented a novel genus within the family *Eggerthellaceae*. Similar results were obtained when the evolutionary relationships between the vaginal isolates and members of the *Eggerthellaceae* were evaluated using the neighbour-joining method (Fig. S1, available in the online Supplementary Material).

## Whole-genome phylogeny and analysis

DNF00809^T^ and PR-HUZ602407-17 cultures (~80 ml) were grown anaerobically for 6 days at 37 °C for genomic DNA extraction using the MasterPure DNA purification kit (Epicenter, Madison, WI) with several modifications to the manufacturer’s protocol as described previously [19]. Single-molecule real-time sequencing was carried out on a Sequel IIe sequencer (Pacific Biosciences, Menlo Park, CA). Sequence assembly was performed with UniCycler v0.4.7 (https://github.com/rrwick/Unicycler/releases), and genomes were annotated using the NCBI Prokaryotic Genome Annotation Pipeline v6.5 [20,22]. CheckM v1.1.3 was used to assess genome completeness and quality [23]. Both DNF00809^T^ and PR-HUZ602407-17 genome sequences were deposited to the NCBI (CP189841 and CP197404). Three contigs were obtained for DNF00809^T^ with a total genome size of 1.78 Mbp, and a single contig was obtained for PR-HUZ602407-17 with a genome size of 1.68 Mbp. CheckM analysis indicated 99% and 98.8% completeness for DNF00809^T^ and PR-HUZ602407-17, respectively, while contamination was indicated at 0% for both isolates.

**Fig. 2.**
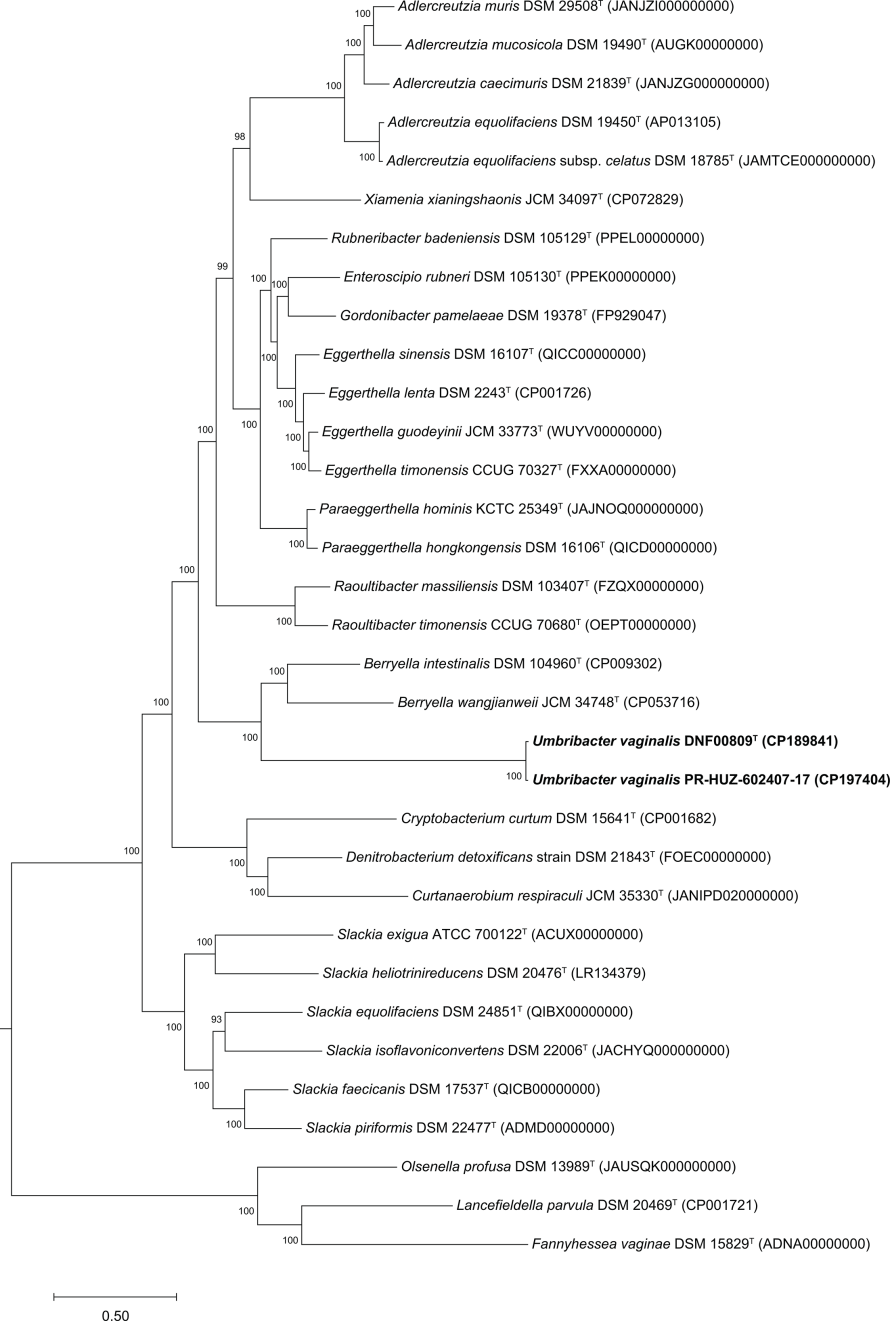
Phylogenomic tree derived from concatenated 100 single-copy marker genes showing the relationship of *U. vaginalis* DNF00809^T^ and *U. vaginalis* PR-HUZ-602407-17 with validly named members of the family *Eggerthellaceae*. Bootstrap values >70% are shown as percentages at each node. Bar, 0.5 substitutions per nt position. *Olsenella profusa* DSM 13989^T^ (JAUSQK000000000), *Lancefieldella parvula* DSM 20469^T^ (CP001721) and *Fannyhessea vaginae* DSM 15829^T^ (ADNA00000000) from the family *Atopobiaceae* were added as the outgroup.

**Fig. 3.**
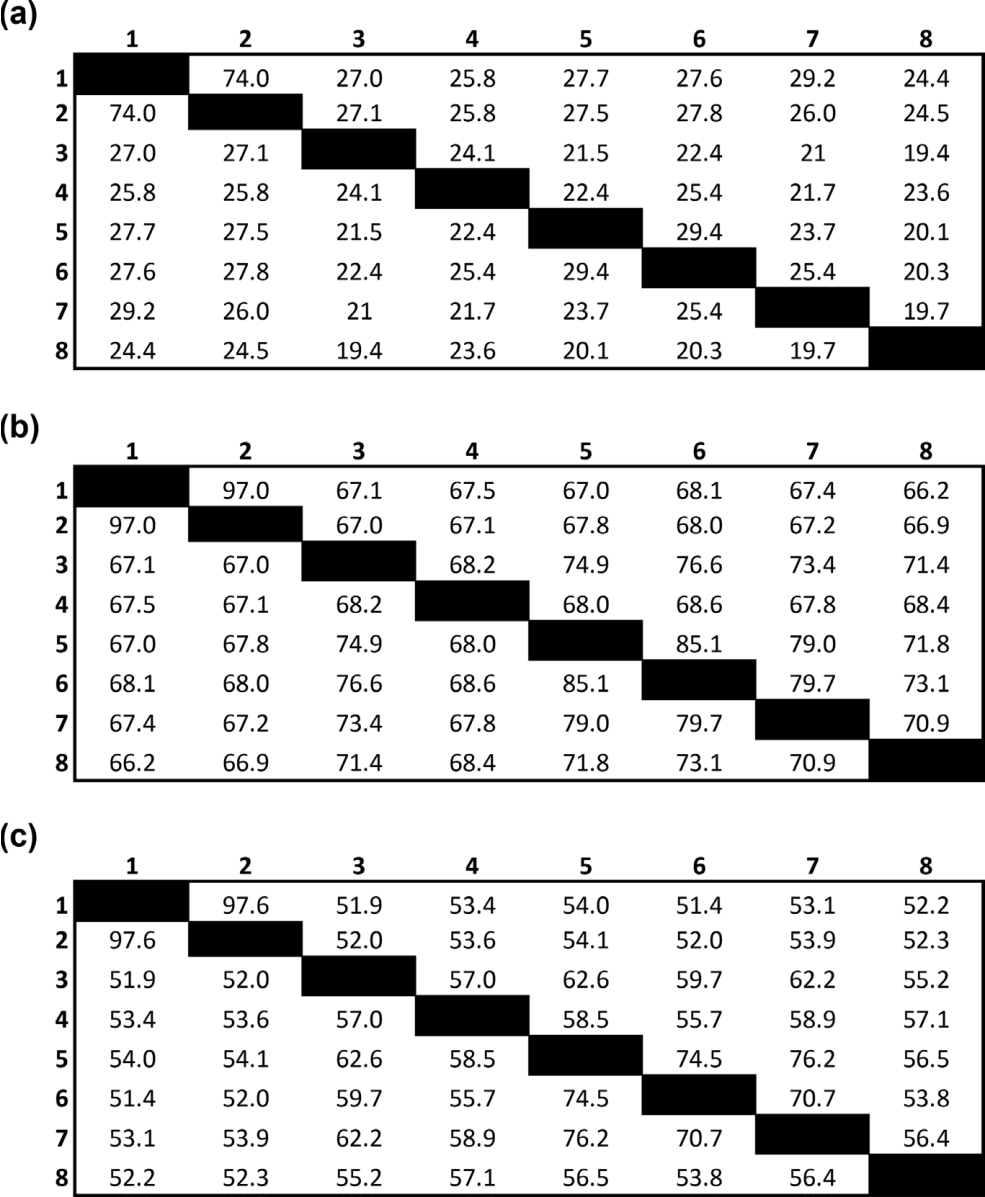
Genome-based species delineation using the (**a**) Genome blast Distance Phylogeny approach. Values less than 70% are indicative of a different species. Strains: 1, *U. vaginalis* DNF00809^T^; 2, *U. vaginalis* PR-HUZ-602407–17; 3, *Adlercreutzia equolifaciens* subsp. *equolifaciens* DSM 19450^T^ [30]; 4, *Cryptobacterium curtum* DSM 15641^T^ [31]; 5, *Eggerthella lenta* DSM 2243^T^ [32]; 6, *Gordonibacter pamelaeae* DSM 19378^T^ [33]; 7, *Paraeggerthella hongkongensis* DSM 16106^T^ [33,34]; 8, *Slackia exigua* DSM 15923^T^ [35,36]. (**b**) ANI analyses. Values of <95% indicate a novel species. (**c**) AAI analyses. Values <65% indicate a novel genus.

A whole-genome phylogenetic tree (Fig. 2) was constructed with genomes of validly named members of the *Eggerthellaceae* using the Codon Tree method with 100 single-copy genes provided in Table S1 using the Bacterial and Viral Bioinformatics Resource Center (BV-BRC) platform 3.25.0 (https://www.bv-brc.org/) [24]. Information regarding DNA G+C content, genome lengths, predicted protein-coding genes and RNA genes was extracted from BV-BRC (Table 1). Digital DNA–DNA hybridization (dDDH), average nt identity (ANI) and average aa identity (AAI) were used to evaluate the relatedness of DNF00809^T^ and PR-HUZ602407-17 to reference genomes from validly named species from the *Eggerthellaceae* family used for comparison in this study. Genome-based species delineation was conducted using Type (Strain) Genome Server (TYGS) with the recommended settings for Formula 2 independent of genome length and robust against incomplete draft genomes (http://tygs.dsmz.de) [25,27]. ANI was calculated using a web-based calculator offered by EzBioCloud (https://www.ezbiocloud.net/tools/ani) [28,29]. AAI was calculated using a web-based tool (https://enveomics.scigap.org/) and two-way AAI values are reported (Fig. 3).

**Table 1.** Comparison of genome characteristics of *U. vaginalis* with selected members of the *Eggerthellaceae* that are validly named

Strains	GenBank accession	Genome length (Mb)	No. of protein coding genes	No. of tRNA genes	No. of rRNA genes	DNA G+C content (mol%)
1	CP189841	1.78	1,614	47	4	46.1
2	CP197404	1.68	1,488	47	4	46.3
3	AP013105.1	2.86	2,324	48	4	63.5
4	CP001682	1.62	1,351	47	6	50.9
5	CP001726	3.63	3,212	48	6	64.2
6	FP929047	3.61	3,083	47	2	60.4
7	QICD00000000	2.80	2,619	54	3	60.9
8	ACUX00000000	2.10	1,813	47	12	62.1

1,* U. vaginalis* DNF00809T; 2,* U. vaginalis* PR-HUZ-602407-17; 3,* Adlercreutzia equolifaciens* subsp. *equolifaciens* DSM 19450T [30]; 4,* Cryptobacterium curtum* DSM 15641T [31]; 5,* Eggerthella lenta* DSM 2243T [32]; 6,* Gordonibacter pamelaeae* DSM 19378T [33]; 7,* Paraeggerthella hongkongensis* DSM 16106T [33,34]; 8,* Slackia exigua* DSM 15923T [35,36].

G+C data was obtained from the genome information provided by the BV-BRC 3.25.0 (https://www.bv-brc.org/) [24].

Representative validly named members of the *Eggerthellaceae* family used for comparisons were *Adlercreutzia equolifaciens* subsp. *equolifaciens* DSM 19450^T^ [30], *Cryptobacterium curtum* DSM 15641^T^ [31], *Eggerthella lenta* DSM 2243^T^ [32], *Gordonibacter pamelaeae* DSM 19378^T^ [33], *Paraeggerthella hongkongensis* DSM 16106^T^ [33,34] and *Slackia exigua* DSM 15923^T^ [35,36]. Isolates were obtained from Leibniz Institute DSMZ-German Collection of Microorganisms and Cell Cultures (DSMZ). Characteristics of the comparison strains were obtained from original manuscripts, and limited testing was performed in this study.

The phylogenomic tree of DNF00809^T^ and PR-HUZ602407-17 and related taxa constructed using 100 core genes showed results similar to the phylogenetic analyses with the 16S rRNA genes (Fig. 2). DNF00809^T^ and PR-HUZ602407-17 clustered closely together, indicating high sequence similarity. Moreover, the 16S rRNA gene sequences for DNF00809^T^ and PR-HUZ602407-17 obtained using the whole genome were 100% identical to the sequences obtained by Sanger sequencing. The dDDH and ANI values of DNF00809^T^ and PR-HUZ602407-17 were 74% and 97.1%, respectively, suggesting that the strains belong to the same species. When compared with other selected members of the *Eggerthellaceae*, the dDDH values of both strains were ≤70% and ANI values were <95% (Fig. 3), indicating that they belong to a novel species [37,38]. The AAI values of both strains were <65% when compared with the other selected members of the *Eggerthellaceae*, suggesting that these strains represent a novel genus [37,39]. The combined results from the phylogenetic, phylogenomic, dDDH, ANI and AAI analyses suggest that DNF00809^T^ and PR-HUZ602407-17 represent a novel genus and species within the *Eggerthellaceae* family.

## Physiology and chemotaxonomy

All bacterial strains were maintained anaerobically on BRU or Wilkins–Chalgren Anaerobe Broth (Thermo Fisher Scientific, Waltham, MA) with 3% sterile heat-inactivated fetal bovine serum (3% FBS-WC) (Corning, NY). An anaerobic atmosphere for all experiments was created using a trimix of 90% N_2_, 5% H_2_ and 5% CO_2_ (Airgas, Radnor, PA). Prior to the use of bacterial isolates in experiments, scrapings from frozen stocks were plated on BRU and incubated anaerobically for at least 72 h at 37 °C. Colonies were sub-cultured twice and examined by Gram staining to ensure purity before use. Bacterial stock cultures used for experiments were verified by 16S rRNA gene sequencing. Isolates were preserved frozen at −80 °C in litmus milk (Becton Dickinson, Franklin Lakes, NJ) or glycerol stocks (10–25% v/v) until testing.

Optimal atmosphere was tested by growing the isolates in aerobic, microaerophilic and anaerobic conditions. DNF00809^T^ and PR-HUZ602407-17 were inoculated onto BRU agar and incubated separately under aerobic conditions, in an AnaeroPack® System or in an AS-580 anaerobic chamber (Anaerobe Systems, Morgan Hill, CA), and with Pouch-MicroAero microaerophilic gas-generating sachet (Mitsubishi Gas Chemical Company, Tokyo, Japan) and box at 37 °C for 7 days. DNF00809^T^ and PR-HUZ602407-17 were both obligate anaerobes. Optimal temperature conditions were tested by growing the isolates on BRU and incubating at different temperatures. DNF00809^T^ grew between 30 and 42 °C (optimal, 30–37 °C) and PR-HUZ602407-17 grew between 25 and 42 °C (optimal, 30–37 °C). Optimal pH was determined by growing the isolates in 3% FBS-WC at a range of 4.5 and 7.5; pH was adjusted with 1M HCl, 1M NaOH or 5M NaOH. Because broth cultures did not become turbid, growth could not be measured by OD. Instead, growth at different pH was assessed by plating 10 µl aliquots of the broth from each pH tube onto BRU at 0, 1, 3, 5 and 7 days of incubation to assess growth semi-quantitatively. DNF00809^T^ showed growth from pH 5.5 to 7.5. PR-HUZ602407-17 exhibited growth between pH 5.5 and 7.5 and delayed growth (at 7 days) at pH 5.0. No growth was noted at pH 4.5; these results are consistent with the high vaginal pH environment (>4.5) noted in BV.

Motility was assessed using 0.3% agar 3% FBS-WC plates with four replicates. Briefly, 2 ml of 3% FBS-WC broth culture for each isolate was centrifuged, and the pellets were resuspended in 200 µl 3% FBS-WC broth. The 0.3% agar plates were stabbed with a P10 pipette tip and then inoculated with the resuspension. The plates were incubated for a total of 10 days. *Escherichia coli* DNF00564 was used as a positive control, and uninoculated WC broth was used as a negative control. For both DNF00809^T^ and PR-HUZ602407-17, growth did not extend throughout the plate and stayed localized to the stab line, indicating they were non-motile (Table 2). Spore formation was assessed in duplicate using methods previously described with 5-day-old broth growth in 3% FBS-WC [40]. *Paeniclostridium sordellii* DSM 2141^T^ was used as a positive control and *E. coli* DNF00564 was used as a negative control. Both DNF00809^T^ and PR-HUZ602407-17 were non-spore-forming (Table 2).

**Table 2.** Comparison of defining characteristics for selected type strains within the *Eggerthellaceae* family

	1	2	3	4	5	6	7	8
% Similarity to *Eggerthella*-like clone 16S rRNA gene sequence (AY738656)	99.9	99.9	92.4	91.2	90.6	91.6	92.1	90.7
**Defining characteristics:**								
Aerotolerance	OA	OA	OA	OA	OA	OA	OA	OA
Cell morphology	C/CB	C/CB	CB	R	R	CB	CB	R
Cell length (µm)	1.08	1.09	1.5–2.7	nd	0.2–2.0	0.8–1.2	nd	1.0
Gram stain	GV	GV	GP	GP	GP	GP	GP	GP
Motility	−	−	−	−	−	+	−	−
Sporulation	−	−	−	−	−	−	−	−
**Presence of:**								
Catalase	w	nd	nd	−	−	+	−	−
Oxidase	−	nd	nd	nd	nd	nd	nd	nd
Nitrate reduction	−	−	−	−	+	−	−	−
**Susceptibility to:**								
Bile, 1 mg	S	nd	S	nd	nd	nd	nd	S
Colistin, 10 µg	R	nd	nd	nd	nd	nd	nd	nd
Vancomycin, 5 µg	S	nd	nd	nd	v	nd	S	nd
Kanamycin, 1000 µg	S	nd	nd	nd	nd	nd	nd	nd
Clindamycin, MIC, µg ml^−1^	0.06, S	nd	nd	nd	nd	nd	nd	nd
Metronidazole, MIC, µg ml^−1^	0.5, S	nd	nd	nd	S	nd	1, S	nd
**Production of:**								
Indole	−	nd	nd	−	nd	nd	−	−
Acetic acid	+	+	−	−	−	−	−	−
Formic acid	+	+	−	−	−	−	−	−
Fumaric acid	+	+	+	−	+	+	+	−
Isobutyric acid	+	+	+	+	+	+	+	−
Lactic acid	−	−	+	+	+	+	+	+
Malonic acid	−	−	−	−	+	+	+	−
Phenylacetic acid	+	+	+	+	+	+	+	+
Propionic acid	+	+	+	+	+	+	+	+
Pyroglutamic acid	+	+	+	+	+	+	+	+
Pyruvic acid	+	+	−	−	−	−	−	−
Reactions to API 32A substrates*								
Arginine arylamidase	+	nd	+	nd	nd	−	v	+
Phenylalanine arylamidase	+	nd	−	nd	nd	−	−	+
Leucine arylamidase	+	nd	+	nd	nd	−	v	+
Tyrosine arylamidase	+	nd	−	nd	nd	−	−	+
Glycine arylamidase	+	nd	−	nd	nd	−	−	+
Histidine arylamidase	+	nd	−	nd	nd	−	−	+
Serine arylamidase	+	nd	−	nd	nd	−	−	+, W
Reactions to API ZYM substrates*								
Esterase (C4)	+	nd	nd	nd	nd	nd	nd	nd
Esterase lipase (C8)	+	nd	nd	nd	nd	nd	nd	nd
Leucine arylamidase	+	nd	nd	nd	nd	nd	nd	nd
Valine arylamidase	+, W	nd	nd	nd	nd	nd	nd	nd
Naphthol-AS-BI-phosphohydrolase	+	nd	nd	nd	nd	nd	nd	nd

1, *U. vaginalis* DNF00809T; 2, *U*. *vaginalis* PR-HUZ-602407-17; 3,* A. equolifaciens* subsp. *equolifaciens* DSM 19450T [30]; 4, *C. curtum* DSM 15641T [31]; 5, *E. lenta* DSM 2243T [32]; 6, *G. pamelaeae* DSM 19378T [33]; 7, *P. hongkongensis* DSM 16106T [33,34]; 8, *S. exigua* DSM 15923T [35,36]. Data for *U. vaginalis* was generated in this study. Comparison data for the other isolates were obtained from original references except for the production of short-chain fatty acids and organic acids which was generated in this study. Nitrate reduction was determined using API Rapid ID 32A. −, Negative; +, positive.

*Only those that are positive for DNF00809T are shown here.

C, cocci; CB, coccobacillus; GP, Gram-positive; GV, Gram variable; nd, not determined; OA, obligately anaerobic; R, resistant; R, rod; S, sensitive; v, variable; W, weak.

**Fig. 4.**
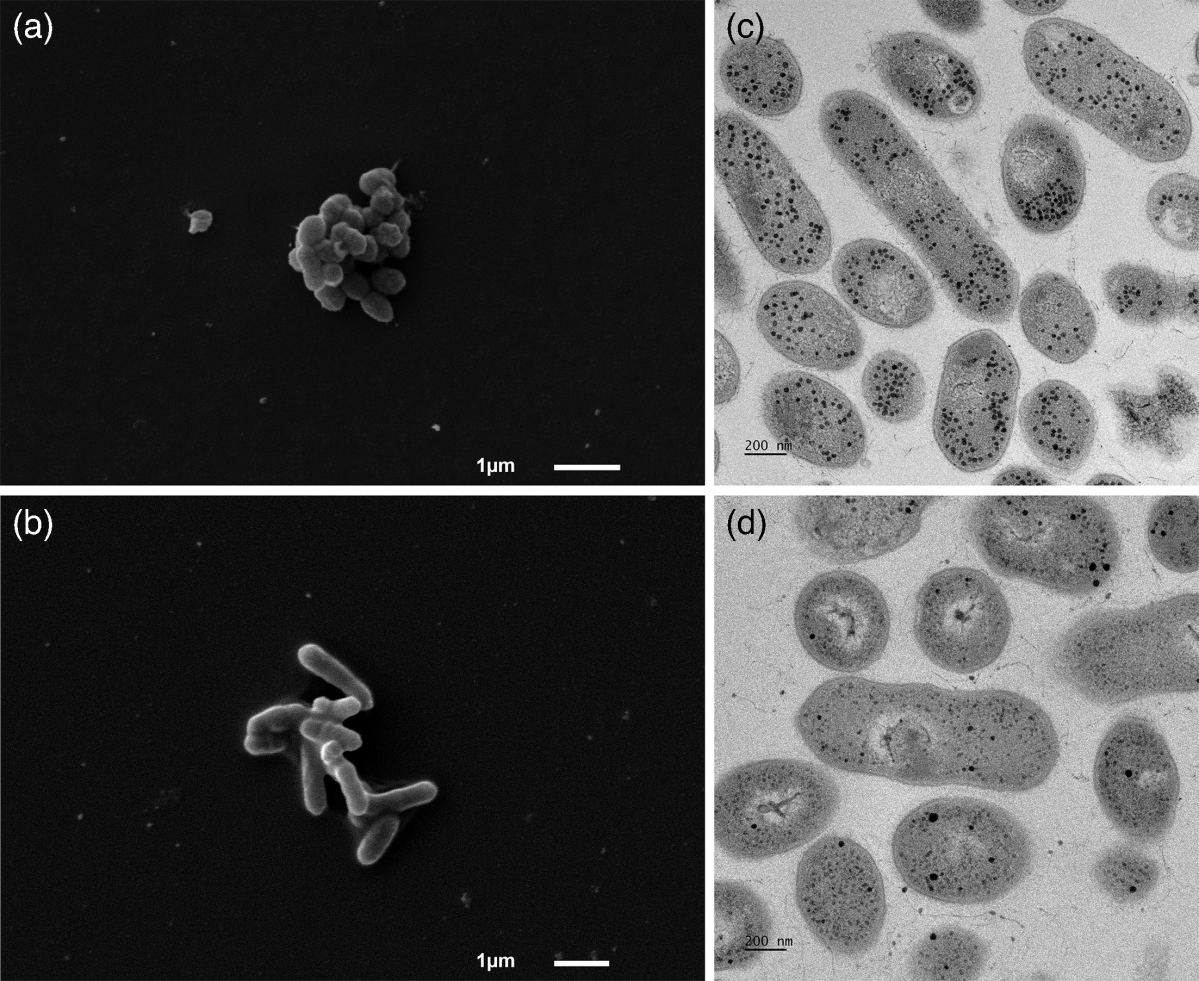
Scanning electron micrographs (bar, 1 µm) of cells of (**a**) *U. vaginalis* DNF00809^T^ and (**b**) *U. vaginalis* PR-HUZ-602407–17. Transmission electron micrograph (bar, 200 nm) of cells of (**c**) *U. vaginalis* DNF00809^T^ and (**d**) *U. vaginalis* PR-HUZ-602407–17. Cells were cultured anaerobically in 3% FBS-WC broth for 6 days at 37 °C.

Colony and cellular morphologies were assessed using a dissecting microscope after DNF00809^T^ and PR-HUZ602407-17 were grown anaerobically at 37 °C for 5 days on BRU. Colonies of both strains DNF00809^T^ and PR-HUZ602407-17 were punctiform, clear, flat and shiny. DNF00809^T^ colonies had an average diameter of 0.4 mm, while colonies of strain PR-HUZ602407-17 had an average diameter of 0.3 mm. Primary examination of Gram staining and cellular morphology was performed with light microscopy at 1,000× magnification. DNF00809^T^ and PR-HUZ602407-17 stained as tiny Gram-variable cocci or short coccobacilli, often in chains. We used the method developed and validated by Halebian *et al*. to distinguish between a Gram-positive and Gram-negative cell wall [41]. Briefly, bacterial colonies are immersed in 3% potassium hydroxide (KOH), and the presence of stringing upon lifting the loop out of the solution is indicative of a Gram-negative cell wall due to disruption and release of DNA threads. Gram-positive bacterial cell walls are less susceptible to degradation in alkaline solutions and stringing will be absent [42]. Due to insufficient growth of the isolates, we were unable to determine the cell wall characteristics with this method. Antibiotic resistance profiles (colistin resistant and vancomycin sensitive) suggest that DNF00809^T^ is Gram-positive which is consistent with the other members of the *Eggerthellaceae* (Table 2). Scanning electron microscopy (SEM) and transmission electron microscopy (TEM) were performed to further evaluate cellular morphology as previously described [43]. Cultures of DNF00809^T^ and PR-HUZ602407-17 (~80 ml each) were grown anaerobically in 3% FBS-WC broth for 6 days at 37 °C and then centrifuged to form a pellet. The pellet was fixed in 0.5 strength Karnovsky’s fixative (2.5% glutaraldehyde, 2% paraformaldehyde in 0.1 M sodium cacodylate buffer, and pH 7.3) overnight at 4 °C for both SEM and TEM. Electron microscopy confirmed the pleomorphic cocci and coccobacillary shape visualized by Gram staining (Fig. 4). DNF00809^T^ cells are on average 1.08 µm in length and 0.52 µm in width, and PR-HUZ602407-17 cells are on average 1.09 µm in length and 0.44 µm in width.

All biochemical testing was performed in duplicate. Initial biochemical tests included catalase (3%, Thermo Fisher Scientific), oxidase (Becton Dickinson) and spot indole (prepared in-house) [42]. DNF00809^T^ exhibited weak catalase activity, did not produce indole and was negative for oxidase (Table 2). API Rapid ID 32A, API ZYM and API 20A (bioMerieux USA, Durham, NC) were completed for DNF00809^T^, and positive reactions to substrates for the ID 32A and ZYM panels can be found in Table 2. DNF00809^T^ was positive for several aminopeptidases including arginine arylamidase and tyrosine arylamidase, indicating they can metabolize peptides. Carbohydrate fermentation was assessed using API 20A enzyme panels instead of pre-reduced anaerobically sterilized peptone-yeast medium (PRAS) with carbohydrate since strains could not reliably be propagated in broth media and growth could not be assessed by OD. All substrate reactions in the API 20A panel were negative, suggesting that DNF00809^T^ is asaccharolytic (data not shown), which is consistent with the comparison of *Eggerthellaceae* strains, *A. equolifaciens* and * S. exigua*, among others [30,36].

The cellular fatty acid composition of DNF00809^T^ and PR-HUZ602407-17 was compared with select type strains from the *Eggerthellaceae* family (Table 2). The fatty acid methyl ester (FAME) data for the comparison strains were obtained from the original references. DNF00809^T^ and PR-HUZ602407-17 were grown on BRU agar and harvested after ~7 days of growth, resuspended in sterile PBS and pelleted by centrifugation. The supernatant was discarded, and pellets were stored at −20 °C and shipped frozen on dry ice to DSMZ, Germany. Analysis of the cellular fatty acids was carried out by DSMZ services, Leibniz-Institute DSMZ – German Collection of Microorganisms and Cell Cultures GmbH, Braunschweig, Germany. GC-MS analysis of fatty acid methyl esters via the traditional sensitive protocol was performed [44]. Both DNF00809^T^ and PR HUZ602407-17 had similar profiles, and the most abundant fatty acids were C_14 : 0_, C_16 : 0_, C_16 : 0_ dimethyl acetal (DMA) and C_18 : 1_*cis*9. While the novel bacteria shared some similarities with other members of the *Eggerthellaceae* family such as an abundance of C_16 : 0_, C_16 : 0_ DMA and C_18 : 1_*cis*9, distinct differences were also noted in abundances and proportions (Table 3).

**Table 3.** Comparison of FAME analysis of novel strains with select organisms within the *Eggerthellaceae* family

Fatty acid composition	1	2	3	5	6	7
12 : 0	2.3	2.3	–	–	–	–
14 : 0	**10.2**	**7.9**	–	–	**8.7**	–
14 : 0 iso	0.6	0.8	2.8	**5.6**	**6.8**	**5.1**
14 : 0 DMA	1.3	1.1	–	–	**9.7**	–
15 : 0 iso	1.4	2	–	–	–	–
15 : 0 anteiso	2.3	**5.5**	–	4	**19.8**	4.2
15 : 0 iso DMA	0.4	0.6	–	–	**6.1**	–
16 : 0	**15.4**	**14.8**	**11.4**	**7.4**	–	**5.7**
16 : 0 DMA	**19.4**	**18.2**	**11.4**	**37.7**	**13.5**	**28.8**
16 : 0 aldehyde	2.4	2.5	2.8	**10.3**	–	**5.7**
17 : 0 DMA	0.4	0.4	–	–	–	2.2
18 : 0	4.5	4.5	**11.4**	**5.3**	–	**5.8**
18 : 0 aldehyde	0.3	0.4	3.6	–	–	2.4
18 : 0 DMA	1.5	1.4	**18.5**	**5.3**	–	**14.1**
18 : 1 *ω*9c	**24.9**	**21.3**	**24.1**	**24.3**	3.7	**20.4**
18 : 1 *ω*11c	2.4	1.5	–	–	–	–
18 : 2 *ω*9, 12 c	1.4	1.9	–	–	–	–
18 : 1 *ω*9c DMA	2.3	3.6	**7.8**	–	–	–
18 : 1 *ω*11c DMA	0.7	0.7	–	–	1.5	2.9
Summed feature 10	–	–	1.2	–	–	2.8

1, *U. vaginalis* DNF00809T; 2, *U*. *vaginalis* PR-HUZ-602407-17; 3, *A. equolifaciens* subsp. *equolifaciens* DSM 19450T [30]; 5, *E. lenta* DSM 2243T [32]; 6, *G. pamelaeae* DSM 19378T [33]; 7, *P. hongkongensis* DSM 16106T [33,34]. Data for *U. vaginalis* was generated in this study. Comparison data were obtained from original references. Data for 4*, C. curtum* DSM 15641T [31], and 8*, S. exigua* DSM 15923T [35,36], are not shown as the original references did not include these data. Summed feature 10 contained one or more of 18 : 1 *ω*11c or 18 : 1 *ω*9t or 18 : 1 *ω*6t. Values are percentages of total fatty acids. Fatty acids >5% are indicated in bold text. If a fatty acid was <1% in all bacteria shown, then it was not included in this table. –, Not detected; DMA, dimethylacetal.

**Fig. 5.**
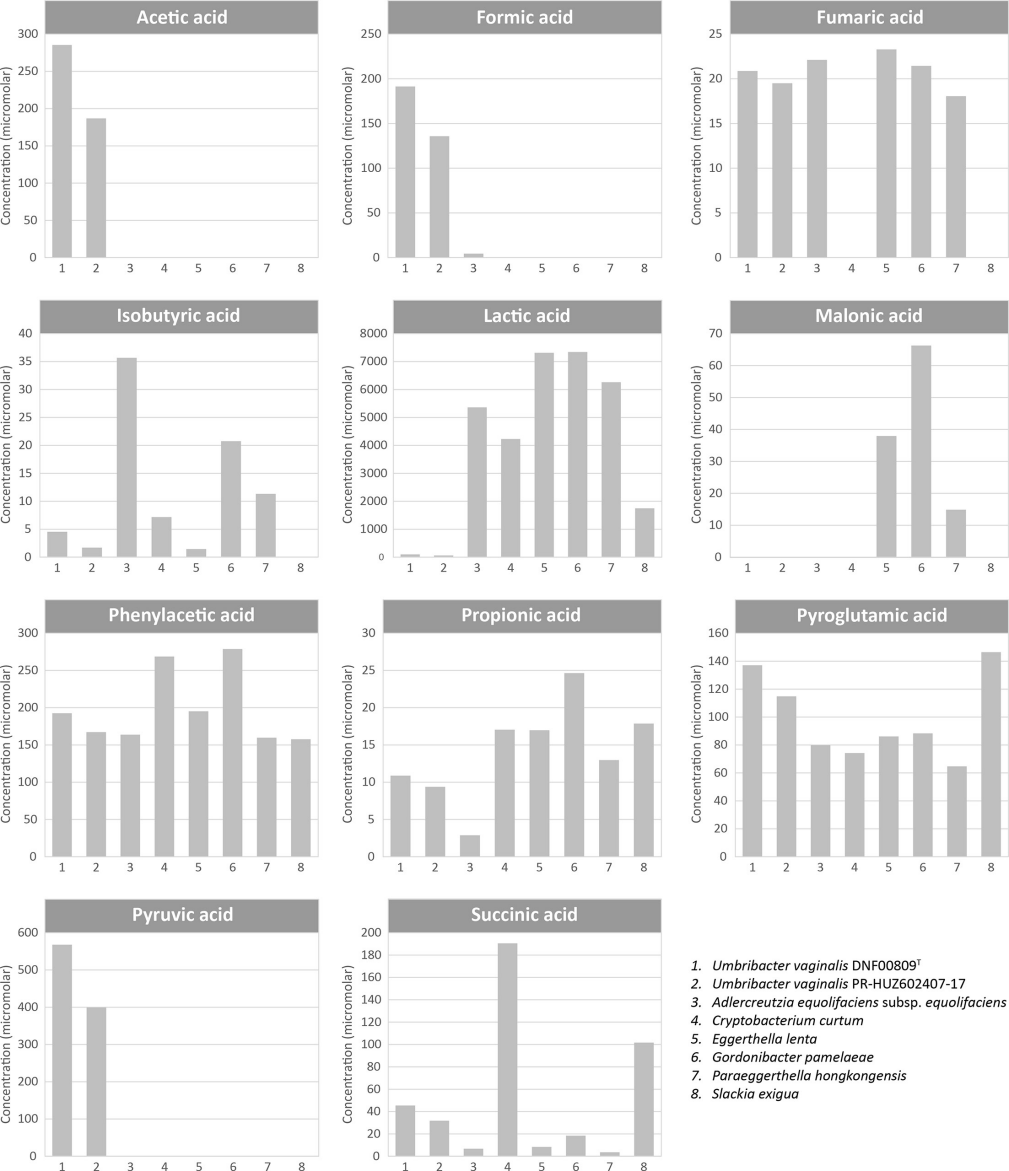
Concentrations of short-chain fatty acids produced after growth of bacterial cells in 3% FBS-WC broth for 72 h at 37 °C. Results are reported as mean values of duplicate experiments, and the overall error is 6.2% between duplicate values. Data for *Umbribacter* and all comparison strains were generated in this study. Concentrations (µM) are shown on the y-axis. 1*, U. vaginalis* DNF00809^T^; 2, *U*. *vaginalis* PR-HUZ-602407–17; 3, *A. equolifaciens* subsp. *equolifaciens* DSM 19450^T^ [30]; 4, *C. curtum* DSM 15641^T^ [31]; 5, *E. lenta* DSM 2243^T^ [32]; 6, *G. pamelaeae* DSM 19378^T^ [33]; 7, *P. hongkongensis* DSM 16106^T^ [33,34]; 8, *S. exigua* DSM 15923^T^ [35,36].

Metabolic end products were measured in both the *Umbribacter* and comparison isolates in duplicate. All isolates were grown in 3% FBS-WC broth for 3 days. Cell supernatants were used for the detection of short-chain fatty acids and organic acids using ^1^H-NMR spectroscopy at the Northwest Metabolomics Research Center at the University of Washington as previously described [43]. In contrast with other *Eggerthellaceae* isolates tested, *Umbribacter* isolates produced acetic acid, formic acid and pyruvic acid, produced very low concentrations of isobutyric acid and did not produce lactic acid or malonic acid (Table 2, Fig. 5). The two *Umbribacter* strains also produced succinic acid, fumaric acid, phenylacetic acid, propionic acid and pyroglutamic acid, similar to other *Eggerthellaceae* isolates (Table 2, Fig. 5). None of the isolates tested produced measurable levels of butyric acid, 2-methyl butyric acid, 2-aminobutyric acid, n-caproic acid, heptanoic acid, isovaleric acid or methylmalonic acid (data not shown).

Antimicrobial susceptibility was performed using the agar dilution method on BRU as per the Clinical and Laboratory Standards Institute (CLSI) for anaerobic bacteria [45]. BV is typically treated with metronidazole or clindamycin [46,49], and susceptibilities to these antibiotics were evaluated for DNF00809^T^ in triplicate (Table 2). Breakpoints for anaerobes for clindamycin are ≤2 µg ml^−1^ (sensitive), 4 µg ml^−1^ (intermediate) and ≥8 µg ml^−1^ (resistant) and for metronidazole, ≤8 µg ml^−1^ (sensitive), 16 µg ml^−1^ (intermediate) and ≥32 µg ml^−1^ (resistant) [45,50]. DNF00809^T^ was susceptible to both metronidazole and clindamycin. Other compounds that we evaluated for activity against DNF00809^T^ included bile (1 mg, prepared in-house), colistin (10 µg, Becton Dickinson), vancomycin (5 µg, Hardy Diagnostics) and kanamycin (1,000 µg, Hardy Diagnostics) [42]. BRU plates were streaked as a lawn across the entire plate, and each disc was placed in the centre of the plate. If the zone of clearance was ≤10 mm, then the isolate was considered sensitive to that compound, while if the zone of clearance was >10 mm, it was considered resistant. DNF00809^T^ was noted to be sensitive to bile, vancomycin and kanamycin, but resistant to colistin (Table 2).

## Description of *Umbribacter* gen. nov.

*Umbribacter* (Um.bri.bac’ter. L. fem. n. *umbra*, ghost or shadow; N.L. masc. n. *bacter*, rod; N.L. masc. n. *Umbribacter*, a ghost-like bacterium).

Cells stain Gram variable, are obligately anaerobic, non-motile, non-spore forming, asaccharolytic and proteolytic. Predominant cell wall fatty acid methyl esters include dimethyl acetals, monounsaturated fatty acids and saturated, unbranched fatty acids. The type species is *U. vaginalis*.

## Description of *Umbribacter vaginalis* sp. nov.

*Umbribacter vaginalis* (va.gi.na’lis. N.L. fem. adj. *vaginalis*, pertaining to the vagina).

Colonies are punctiform, clear like a water droplet, flat and shiny with a diameter of 0.3–0.4 mm following 5 days anaerobic incubation at 37 °C on Brucella agar supplemented with 5% laked sheep blood, hemin and vitamin K. Growth is similar on Wilkins–Chalgren Anaerobe Agar with 3% sterile heat-inactivated fetal bovine serum with colonies appearing as punctiform, clear, flat and shiny after at least 5 days of anaerobic incubation. Once the colony size of 0.3–0.4 mm is achieved, additional incubation does not result in an increase in colony size. Cells are non-motile, non-spore forming, stain as Gram-variable cocci and coccobacilli and are ~1.08–1.09 µm in length and 0.44–0.52 µm in width. The bacterium is sensitive to vancomycin, kanamycin and bile, resistant to colistin, weakly positive for catalase activity and negative for oxidase activity and nitrate reduction and does not produce indole. Optimal temperature for growth ranges between 30 and 37 °C. Asaccharolytic, with enzymatic activities present for arginine arylamidase, phenylalanine arylamidase, leucine arylamidase, valine arylamidase, esterase, esterase lipase and naphthol-AS-BI-phosphohydrolase. Major metabolic end products produced are acetate, formate, fumarate, phenylacetate, propionate, pyruvate, pyroglutamate and succinate. Susceptible to clindamycin and metronidazole. Predominant cell wall fatty acids are C_14 : 0_, C_16 : 0_, C_16 : 0_ dimethyl acetal and C_18 : 1_*cis*9.

The type strain is DNF00809^T^ (=DSM 118866^T^=CCUG 77988^T^), which was isolated from a vaginal fluid sample from a woman with BV. The DNA G+C content is 46.1 mol%. Accession numbers of the 16S rRNA gene sequence and the whole-genome sequence are KP192306 and CP189841, respectively.

## Supplementary material

10.1099/ijsem.0.006931 Uncited Supplementary Material 1.

## Abbreviations

AAI: average aa identity
ANI: average nt identity
BV: bacterial vaginosis
BV-BRC: Bacterial and Viral Bioinformatics Resource Center
dDDH: digital DNA–DNA hybridization
ELVB: *Eggerthella*-like vaginal bacterium
FAME: fatty acid methyl ester
HIV: human immunodeficiency virus
PID: pelvic inflammatory disease
SEM: scanning electron microscopy
TEM: transmission electron microscopy
