# Supplementary material for: Umbribacter vaginalis gen. nov., sp. nov.: novel bacterium isolated from the human vagina
Source: Int J Syst Evol Microbiol. 2025 Oct 3;75(10):006931. doi: 10.1099/ijsem.0.006931 (PMC12494484; doi:10.1099/ijsem.0.006931)
Supplement: Uncited Supplementary Material 1. [file ijsem-75-06931-s001.pdf]

SUPPLEMENTARY FIGURE LEGEND

Supplementary Fig. 1. Molecular phylogenetic analysis by neighbor joining method on 16S rRNA gene sequences showing the phylogenetic positions of *Umbribacter vaginalis* DNF00809^{T} and *U. vaginalis* PR-HUZ-602407-17 in comparison with members of the family *Eggerthellaceae*. Bootstrap values (based on 1000 replications) greater than or equal to 70% are shown as percentages at each node. Bar, 0.02 substitutions per nucleotide position. *Olsenella profusa* DSM 13989^{T} (AF292374), *Lancefieldella parvula* DSM 20469^{T} (CP001721) and *Fannyhessea vaginae* DSM 15829^{T} (ACGK00000000.2) from the family *Atopobiaceae* were added as the outgroup.

Supplementary Figure 1

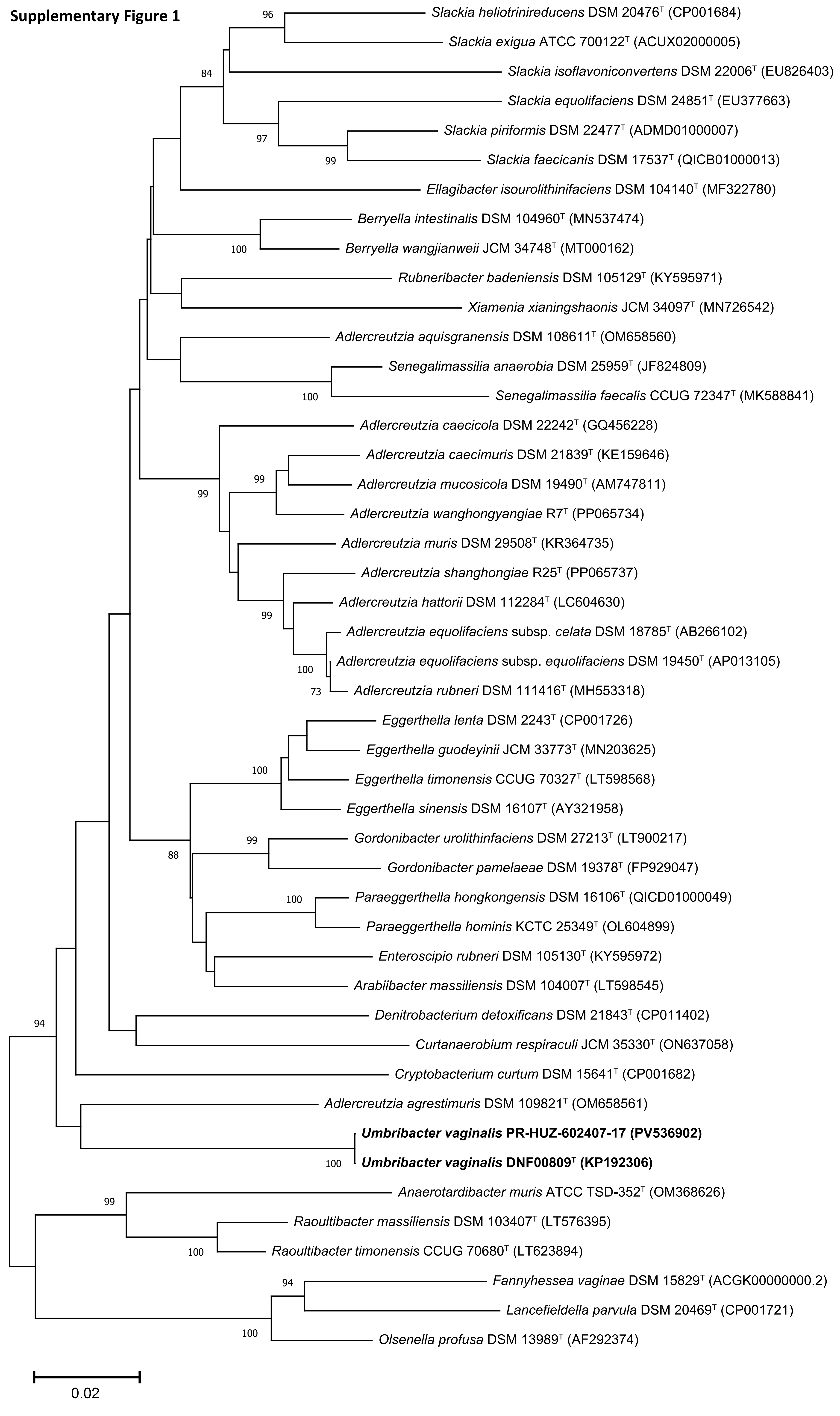

Supplementary Table 1. List of single copy genes used to construct the phylogenomic tree using the Codon Tree method.

	PGFam	Product		PGFam	Product		PGFam	Product		PGFam	Product
1	PGF_02704551	DNA-directed RNA polymerase beta' subunit (EC 2.7.7.6)	26	PGF_00134118	Predicted insulinase-like Zn-dependent peptidase DVU0941	51	PGF_08155727	DNA translocase FtsK	76	PGF_00016431	LSU ribosomal protein L3p (L3e)
2	PGF_00045999	Pyruvate,phosphate dikinase (EC 2.7.9.1)	27	PGF_00052238	Signal recognition particle protein Ffh	52	PGF_10376398	Peptidoglycan D,D-transpeptidase MrdA (EC 3.4.16.4)	77	PGF_02959749	Ribonuclease D (EC 3.1.26.3)
3	PGF_03999196	ATP-dependent Clp protease, ATP-binding subunit ClpC	28	PGF_00423429	Dihydroxyacetone kinase-like protein, phosphatase domain / Dihydroxyacetone kinase-like protein, kinase domain	53	PGF_00024478	NAD-dependent glyceraldehyde-3-phosphate dehydrogenase (EC 1.2.1.12)	78	PGF_07420523	Exodeoxyribonuclease III (EC 3.1.11.2)
4	PGF_00060409	Translation elongation factor G	29	PGF_00020781	Methionyl-tRNA synthetase-related protein 2	54	PGF_00011081	Helicase PriA essential for oriC/DnaA-independent DNA replication	79	PGF_00049904	SSU ribosomal protein S7p (S5e)
5	PGF_00950554	Excinuclease ABC subunit B	30	PGF_01175575	Threonyl-tRNA synthetase (EC 6.1.1.3)	55	PGF_06759833	Peptide chain release factor 2	80	PGF_06857975	Cell-division-associated, ABC-transporter-like signaling protein FtsE
6	PGF_05500127	Valyl-tRNA synthetase (EC 6.1.1.9)	31	PGF_00037588	Prolyl-tRNA synthetase (EC 6.1.1.15), bacterial type	56	PGF_00048782	Ribose-phosphate pyrophosphokinase (EC 2.7.6.1)	81	PGF_00425738	Sporulation transcription regulator WhiA
7	PGF_07830674	Alanyl-tRNA synthetase (EC 6.1.1.7)	32	PGF_00063916	Tyrosyl-tRNA synthetase (EC 6.1.1.1)	57	PGF_00275905	Glutamine synthetase type I (EC 6.3.1.2)	82	PGF_00048846	Ribosomal protein S12p Asp88 (E. coli) methylthiotransferase (EC 2.8.4.4)
8	PGF_06703483	DNA gyrase subunit B (EC 5.99.1.3)	33	PGF_00926109	Arginyl-tRNA synthetase (EC 6.1.1.19)	58	PGF_05767868	Probable transcriptional regulatory protein YebC	83	PGF_02923127	Uridylate kinase (EC 2.7.4.22)
9	PGF_00060414	Translation elongation factor LepA	34	PGF_00422271	DNA-directed RNA polymerase alpha subunit (EC 2.7.7.6)	59	PGF_00033359	Phospho-N-acetylmuramoyl-pentapeptide-transferase (EC 2.7.8.13)	84	PGF_10381714	Orotate phosphoribosyltransferase (EC 2.4.2.10)
10	PGF_00007041	GTP-binding protein TypA/BipA	35	PGF_00051525	Seryl-tRNA synthetase (EC 6.1.1.11)	60	PGF_00049889	SSU ribosomal protein S3p (S3e)	85	PGF_10569727	LSU rRNA pseudouridine(1911/1915/1917) synthase (EC 5.4.99.23)
11	PGF_05171623	Isoleucyl-tRNA synthetase (EC 6.1.1.5)	36	PGF_00007024	GTP-binding protein EngA	61	PGF_02390924	16S rRNA (cytosine(1402)-N(4))-methyltransferase (EC 2.1.1.199)	86	PGF_00015259	ATP synthase gamma chain (EC 3.6.3.14)
12	PGF_05195027	ATP synthase beta chain (EC 3.6.3.14)	37	PGF_00007012	GTP-binding and nucleic acid-binding protein YchF	62	PGF_08454293	N-acetylglucosamine-1-phosphate uridyltransferase (EC 2.7.7.23) / Glucosamine-1-phosphate N-acetyltransferase (EC 2.3.1.157)	87	PGF_05621995	Prolipoprotein diacylglyceryl transferase
13	PGF_00033095	Phenylalanyl-tRNA synthetase beta chain (EC 6.1.1.20)	38	PGF_00016393	LSU ribosomal protein L2p (L8e)	63	PGF_00426236	(E)-4-hydroxy-3-methylbut-2-enyl-diphosphate synthase (flavodoxin) (EC 1.17.7.3)	88	PGF_04127376	Twin-arginine translocation protein TatC
14	PGF_07058357	Single-stranded-DNA-specific exonuclease RecJ	39	PGF_02029783	GTP-binding protein Obg	64	PGF_00062045	Tryptophanyl-tRNA synthetase (EC 6.1.1.2)	89	PGF_00016443	LSU ribosomal protein L5p (L11e)
15	PGF_04569524	ATP synthase alpha chain (EC 3.6.3.14)	40	PGF_00047078	RecA protein	65	PGF_00057483	Transcription termination factor Rho	90	PGF_03174068	Transcription antitermination protein NusG
16	PGF_00060428	Translation elongation factor Tu	41	PGF_00030640	Peptide chain release factor 1	66	PGF_06115957	tRNA 4-thiouridine synthase (EC 2.8.1.4)	91	PGF_00049837	SSU ribosomal protein S11p (S14e)
17	PGF_00013003	Bis-ABC ATPase YheS	42	PGF_00900122	UDP-N-acetylglucosamine 1-carboxyvinyltransferase (EC 2.5.1.7)	67	PGF_10505717	Glucose-6-phosphate isomerase (EC 5.3.1.9)	92	PGF_00007027	GTP-binding protein Era
18	PGF_10347756	DNA polymerase I (EC 2.7.7.7)	43	PGF_00423378	Dihydroorotase (EC 3.5.2.3)	68	PGF_00009967	Glycyl-tRNA synthetase alpha chain (EC 6.1.1.14)	93	PGF_07695531	LSU ribosomal protein L11p (L12e)
19	PGF_05581732	Protein translocase subunit SecY	44	PGF_00423472	DinG family ATP-dependent helicase YoaA	69	PGF_08932911	Alanine racemase (EC 5.1.1.1)	94	PGF_00016343	LSU ribosomal protein L16p (L10e)
20	PGF_03000099	Ribonuclease Y	45	PGF_03029859	Bis-ABC ATPase Uup	70	PGF_06936561	Translation elongation factor Ts	95	PGF_00033289	Phosphate:acyl-ACP acyltransferase PlsX (EC 2.3.1.n2)
21	PGF_01054379	Adenylosuccinate lyase (EC 4.3.2.2) @ SAICAR lyase (EC 4.3.2.2)	46	PGF_05049118	Holliday junction ATP-dependent DNA helicase RuvB (EC 3.6.4.12)	71	PGF_03701810	Cell-division-associated, ABC-transporter-like signaling protein FtsX	96	PGF_03799365	Translation elongation factor P
22	PGF_03609651	Methionyl-tRNA synthetase (EC 6.1.1.10)	47	PGF_00008337	Glutamyl-tRNA synthetase (EC 6.1.1.17) @ Glutamyl-tRNA(Gln) synthetase (EC 6.1.1.24)	72	PGF_04244475	Adenylate kinase (EC 2.7.4.3)	97	PGF_04512522	LSU ribosomal protein L4p (L1e)
23	PGF_00024831	NADP-specific glutamate dehydrogenase (EC 1.4.1.4)	48	PGF_02061452	Queuine tRNA-ribosyltransferase (EC 2.4.2.29)	73	PGF_01126005	Rod shape-determining protein RodA	98	PGF_00016444	LSU ribosomal protein L6p (L9e)
24	PGF_06935032	Adenylosuccinate synthetase (EC 6.3.4.4)	49	PGF_00066906	Aspartate carbamoyltransferase (EC 2.1.3.2)	74	PGF_00049362	S-adenosylmethionine:tRNA ribosyltransferase-isomerase (EC 2.4.99.17)	99	PGF_04991657	Oxygen-independent coproporphyrinogen-III oxidase-like protein YggW
25	PGF_06848757	Ribonuclease J2 (endoribonuclease in RNA processing)	50	PGF_08562657	tRNA-i(6)A37 methylthiotransferase (EC 2.8.4.3)	75	PGF_02895613	Methylenetetrahydrofolate--tRNA-(uracil-5-)-methyltransferase TrmFO (EC 2.1.1.74)	100	PGF_06941403	SSU ribosomal protein S12p (S23e)
